# Supplementary material for: A Convenient Oligonucleotide Conjugation via Tandem Staudinger Reaction and Amide Bond Formation at the Internucleotidic Phosphate Position
Source: Int J Mol Sci. 2024 Feb 7;25(4):2007. doi: 10.3390/ijms25042007 (PMC10889076; doi:10.3390/ijms25042007)
Supplement: Supplementary file 1 [file ijms-25-02007-s001.zip › ijms-2793242-supplementary.pdf]

## Supplementary Material

### General information

All reactions were carried out under argon atmosphere using anhydrous solvents unless otherwise stated. Standard *N*-protected deoxyribonucleoside 5′-DMTr-3′-β-cyanoethyl-*N*,*N*-diisopropyl phosphoramidites (Sigma-Aldrich Inc., St Louis, MO, USA) and 500Å CPG polymer supports (Glen Research Corp, Sterling, VA, USA) were used for oligonucleotide synthesis. Acetonitrile (UHPLC Supergradient, Panreac, Spain) for oligonucleotide synthesis was refluxed over $CaH_2$ under argon for 6 h, distilled and stored under argon over 3Å molecular sieves. All amines used for conjugation were from Sigma-Aldrich (Saint Louis, MO, USA). For HPLC, Supergradient UHPLC grade acetonitrile (Panreac, Madrid, Spain) and triethylammonium acetate (TEAA) buffer, pH 7.0 (Thermo Fisher Scientific, USA) were used. Stains-All, Xylene Cyanol FF, and Bromophenol Blue (BP) dyes together with sodium cacodylate were from Sigma-Aldrich (Saint Louis, MO, USA), sodium perchlorate and *N*,*N*′-dicyclohexylcarbodiimide from Acros Organics (Carlsbad, CA, USA), dichloromethane, tetrahydrofuran and pyridine from Panreac (Madrid, Spain). Formamide, acrylamide, *N*,*N*′-methylene-*bis*-acrylamide, urea, *tris*(hydroxymethyl)-aminomethane (Tris), boric acid, disodium ethylenediaminetetraacetate ($Na_2EDTA$) were from Dia-M (Moscow, Russia). Conc. aq. ammonia solution and acetone (purest grade) were from SoyuzKhimProm (Novosibirsk, Russia). Reagents and solvents were purchased from their respective commercial suppliers, and used without further purification unless otherwise stated. Bi-distilled water was prepared in the laboratory.

For centrifugation of small volumes, a MiniSpin Plus microcentrifuge (Eppendorf, Hamburg, Germany) was used. Chemical reactions are carried out using a Thermomixer Compact thermoshaker (Eppendorf, Hamburg, Germany). The solutions were shaken using a BioVortex V1 vortex (Biosan, Riga, Latvia). Gel electrophoresis was carried out using an electrophoresis unit from Bio-Rad (Hercules, CA, USA). Small volumes of oligonucleotide solutions up to 1.5 mL were evaporated in a Concentrator Plus vacuum concentrator (Eppendorf, Hamburg, Germany). Oligonucleotide solutions, after purification, were lyophilized using a FreeZone freeze-drier (Labconco, Kansas City, MO, USA). Mass spectra were recorded using a MALDI-TOF Autoflex II LRF50-CID mass spectrometer (Bruker Daltonics, Germany). The optical densities of the solutions of oligonucleotide conjugates were measured using a NanoPhotometer NP80-Touch (Implen, Germany). Thermal denaturation curves were recorded on a UV-1800 UV-VIS spectrophotometer (Shimadzu, Japan) equipped with Peltier unit.

$^{1}$H NMR spectra were recorded in solution (20–40 mg/mL) on Bruker DRX-500, Avance 400, or Avance 300 spectrometers (500, 400, and 300 MHz, respectively). $^{13}$C NMR spectra were acquired on Bruker DRX-500 or Avance 400 spectrometers (125 and 100 MHz, respectively). The residual solvent signals were used as internal standards for $^{1}$H and $^{13}$C NMR ($CDCl_3$: 7.24 ppm for $^{1}$H, 76.9 ppm for $^{13}$C; DMSO-$d_6$: 2.50 ppm for $^{1}$H, 39.5 ppm for $^{13}$C). The assignment of signals in the $^{13}$C NMR spectra was done based on calculations within the framework of the DFT ($CHCl_3$ as the solvent). Signal assignment was made using J modulated $^{13}$C NMR spectra (proton-noise decoupling, the opposite phases for the signals of the atoms with the odd and even numbers of the attached protons, tuning to the constant $J$ = 135 Hz) and 2D NMR spectra: 1) homonuclear $^{1}$H $^{1}$H correlation, 2) heteronuclear $^{13}$C $^{1}$H correlation at the direct spin-spin coupling constants ($J$ = 135 Hz), 3) heteronuclear $^{13}$C $^{1}$H correlation at the long range spin-spin coupling constants ($J$ = 10 Hz), and 4) homonuclear J-resolved $^{1}$H spectra. Carbon-proton spin-spin coupling constants were taken from proton-coupled $^{13}$C NMR spectra. Sign of spin-spin couplings was not determined. Chemical shifts (  values) are expressed in ppm, coupling constants ($J$) are expressed in Hz, and multiplicities are mentioned as follows: s (singlet), d (doublet), t (triplet), q (quartet), m (multiplet). Width at half-height ($W_{1/2}$) for broadened signals is given in Hz. Critical resolution parameters when registering 1D NMR spectra: spectral width 5500 Hz (11 ppm) for $^{1}$H and 31.44 KHz (250 ppm) for $^{13}$C, size of FID 16K for $^{1}$H and 32K for $^{13}$C, size of real spectrum 128K for both $^{1}$H and $^{13}$C, spectral resolution 0.042 Hz for $^{1}$H and 0.24 Hz (0.002 ppm) for $^{13}$C.

Mass spectra were recorded on a Thermo Electron DFS spectrometer (EI ionization, 70 eV). Elemental analysis was performed on an EA 3000 CHNS-analyzer, HEKAtech GmbH (Germany). Merck silica gel (63–100 μ) was used for flash chromatography. Monitoring of the reaction progress was done by taking $^{1}$H NMR spectra of the reaction mixture, and by TLC on Sorbfil plates (visualization by iodine vapors or UV irradiation).

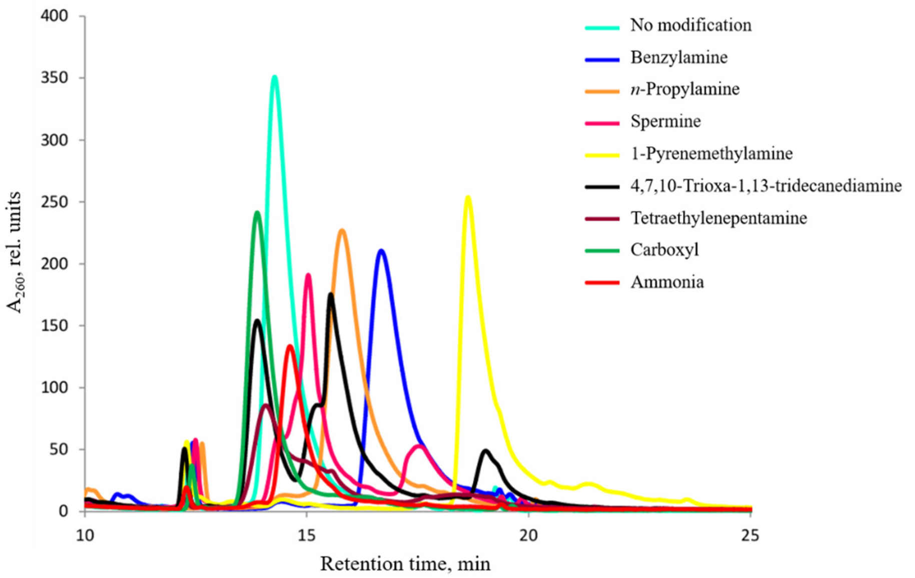


**Figure S1**. An overlap of elution profiles of crude reaction mixtures from the syntheses of 5′-singly-modified 17-mer oligonucleotides 5′-A$^{\xi}$GTCTCGACTTGCTACC obtained by **A**/HBTU/HOBt method (Table 1).

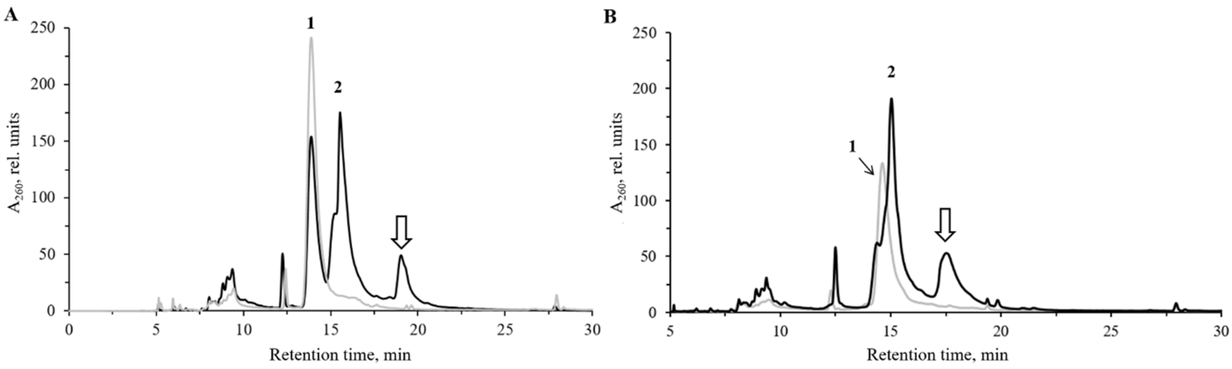


**Figure S2**. Elution profiles of the conjugates of 5′-A$^{\xi}$GTCTCGACTTGCTACC obtained by **A**/HBTU/HOBt method: (**A**) with 4,7,10-trioxa-1,13-tridecanediamine; traces (**1**) – carboxyl oligonucleotide (**A6**), and (**2**) – conjugate (**A16**); and (**B**) with spermine; traces (**1**) amide oligonucleotide (**A7**), and (**2**) conjugate. By-products resulting from acrylonitrile addition are marked by white arrows (see [82]).

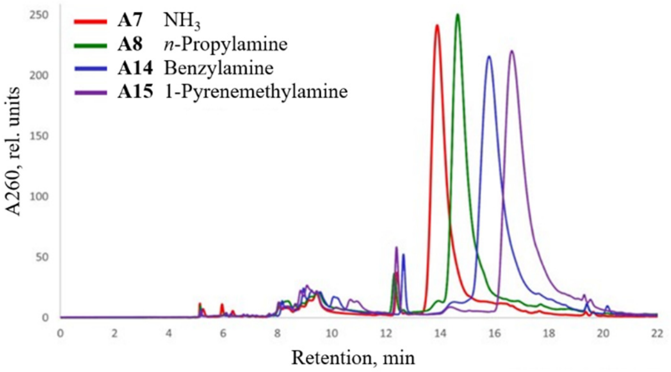


**Figure S3**. RP-HPLC profiles of crude oligonucleotide conjugates **A7**, **A8**, **A14**, **A15**; elution gradient (*ii*) (Section 4).

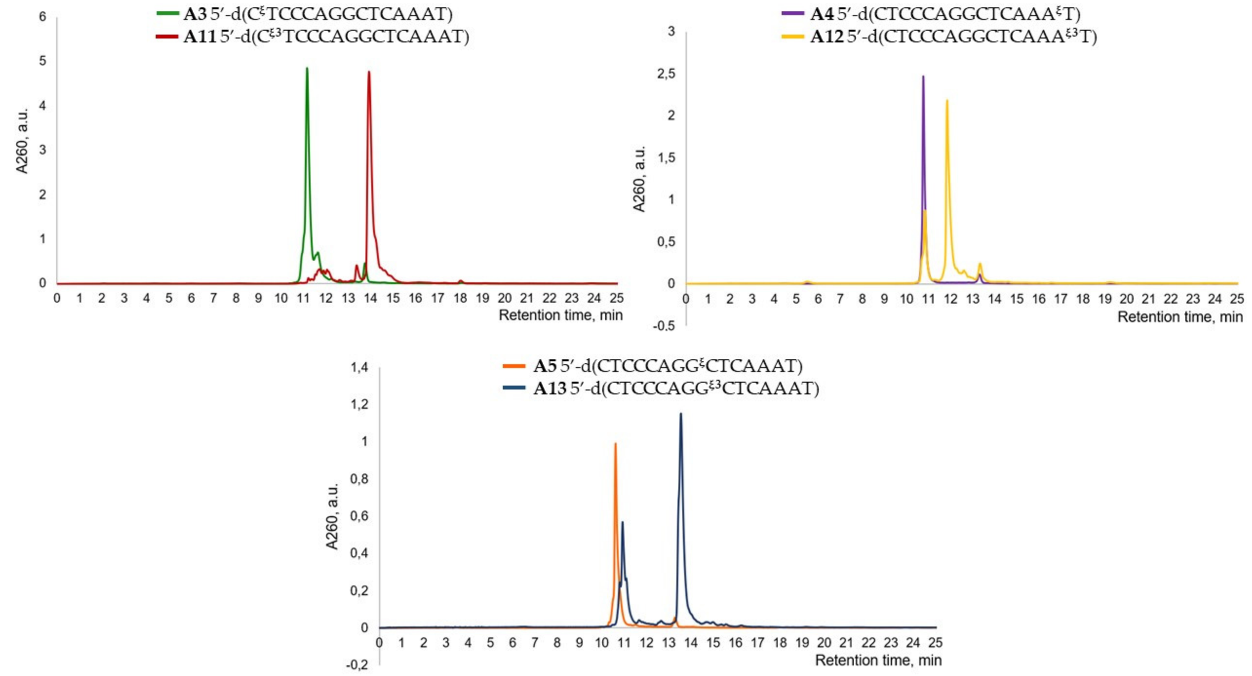


**Figure S4**. RP-HPLC profiles of crude modified oligonucleotides **A3-A5** and their conjugates with benzylamine **A11-A13** (Table 1).

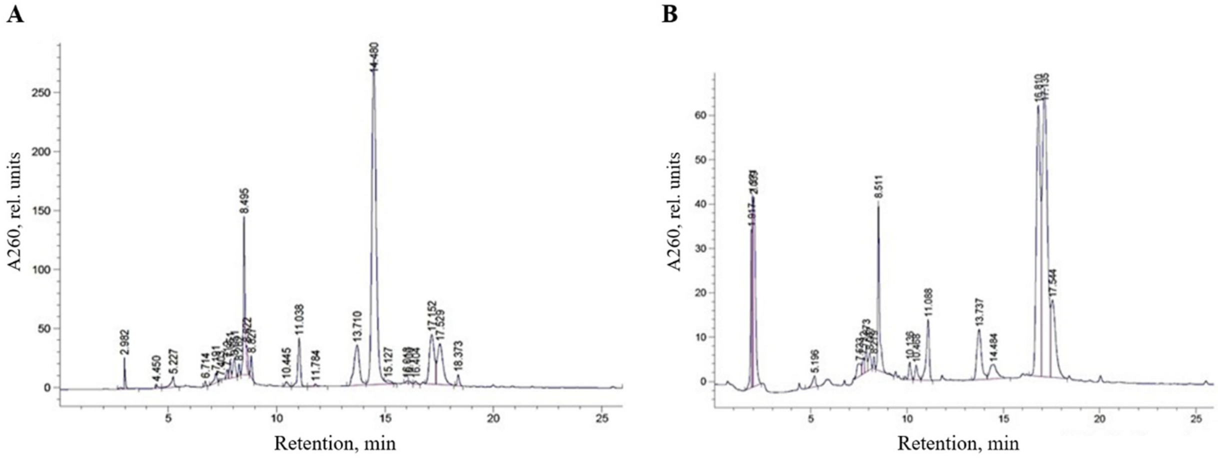


**Figure S5**. RP-HPLC profiles of crude oligonucleotide conjugates: (**A**) **C1**; and (**B**) **C2** (Table 1).

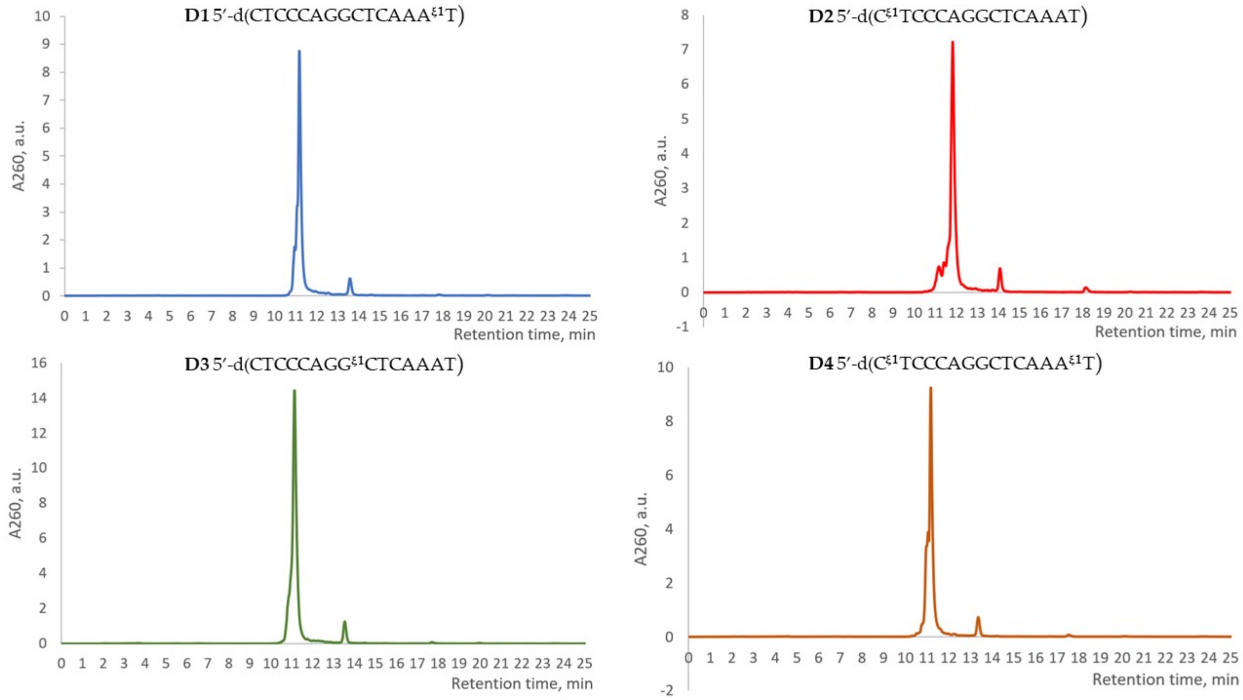


**Figure S6**. RP-HPLC profiles of crude oligonucleotide conjugates **D1**-**D4** (Table 1).

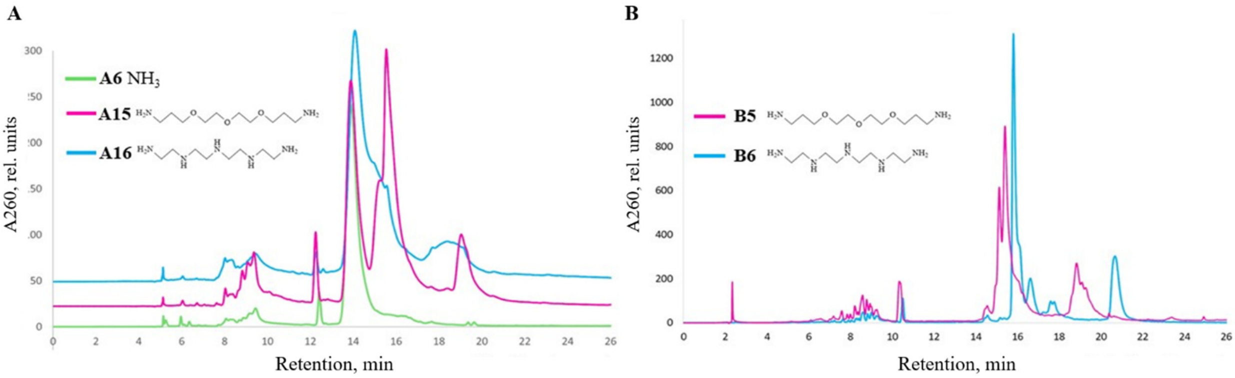


**Figure S7**. RP-HPLC profiles of crude oligonucleotide conjugates: (**A**) **A6**, **A15**, and **A16**; and (**B**) **B5** and **B6** (Table 1).

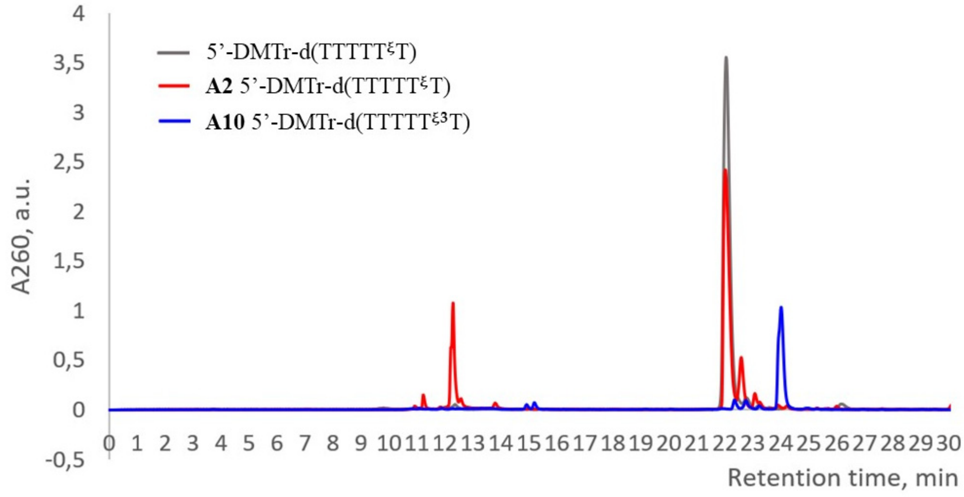


**Figure S8**. RP-HPLC elution profiles of crude oligonucleotide **A2** and its conjugate with benzylamine **A10** (Table 1).

**Table S1**. MALDI TOF MS of oligonucleotide conjugates.

| Oligonucleotide sequence, 5′-3′ | | Molecular mass, Da[a] | | |
|---|---|---|---|---|
| | | Calc. [M-H]⁻ | Exp. [M-H]⁻ | |
| d(A$^{\xi}$GTCTCGACTTGCTACC) | | 5606.02 | 5604.78 | |
| | | | **A** | **B** |
| **1** | $NH_3$ | 5604.03 (5301.03[b]) | 5604.78 | 5301.65[b] |
| **2** | *n*-Propylamine | 5646.98 (5344.61[b]) | 5647.91 (5344.75[b]) | 5647.60 (5345.36[b]) |
| **3** | Benzylamine | 5695.02 (5392.65[b]) | 5391.86 | 5694.89 (5391.32[b]) |
| **4** | 1-Pyrenemethylamine | 5819.17 | 5819.86 | 5818.35 |
| **5** | 4,7,10-Trioxa-1,13-tridecanediamine | 5808.17 | – | 5806.47 |
| **6** | Tetraethylenepentamine | 5777.17 | 5777.71 | 5775.41 |

[a] Letters **A** and **B** indicate the corresponding azideы from which the modified oligonucleotides were obtained; Nos. **1**–**6** indicate the amines used in conjugation reaction; [b] Molecular masses of DMTr OFF oligonucleotides; DMTr – 4,4′-dimethoxytrityl.

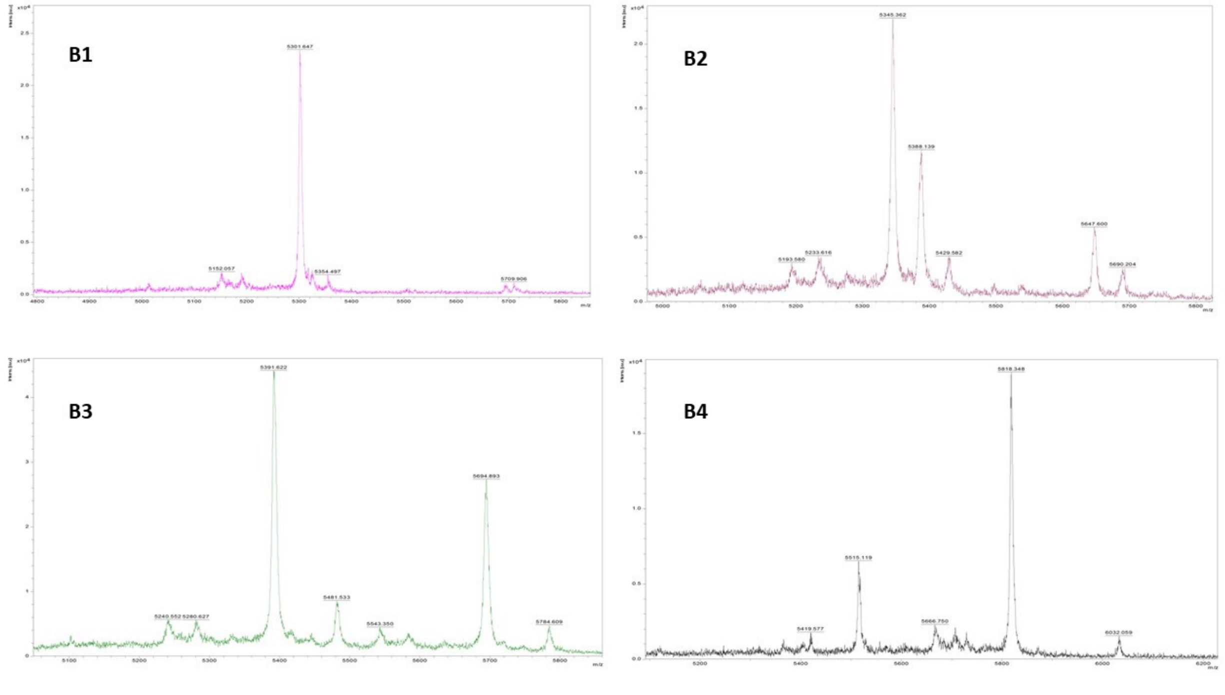


**Figure S9**. MALDI-TOF MS analysis of oligonucleotide conjugates **B1-B4**.

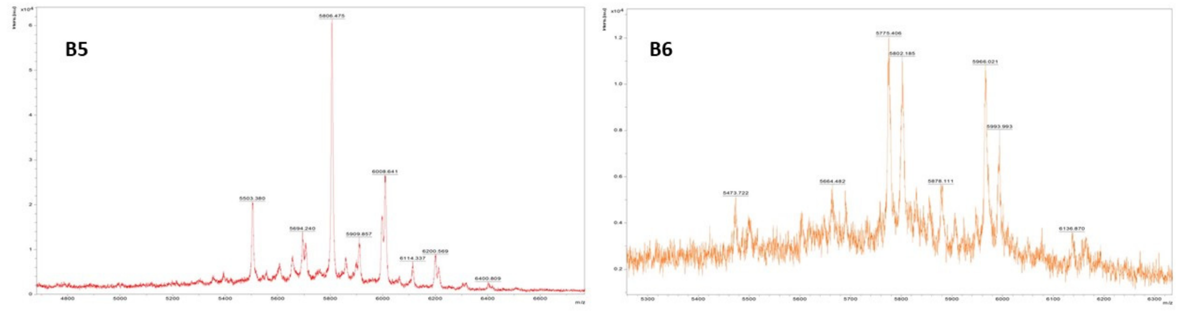


**Figure S10**. MALDI-TOF MS spectra of oligonucleotide conjugates **B5-B6** (see Table 1).

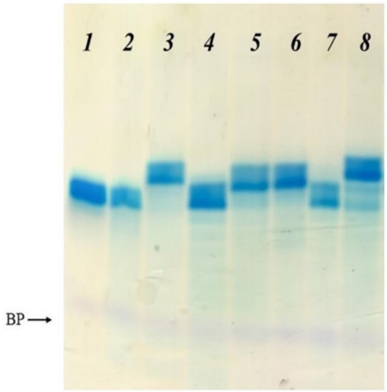


**Figure S11**. Electrophoretic comparison of the mobility of modified oligonucleotides with ξ-group and their conjugates with benzylamine: lanes: (*1*) – unmodified 15-mer control, (*2*) – **A3**, (*3*) – **A11**, (*4*) – **A4**, (*5*) and (*6*) – **A12**, (*7*) – **A5**, and (*8*) – **A13**. BP – Bromophenol Blue.

## Synthesis of pentafluorophenyl (B), 4-nitrophenyl (C), and pentafluorobenzyl (D) esters of 4-(azidosulfonyl)-benzoic acid

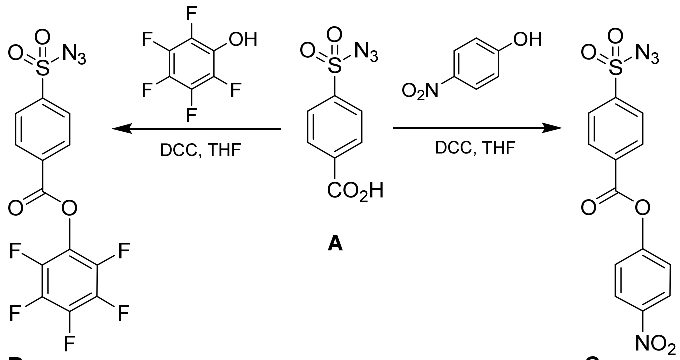


**Figure S12**. Synthesis of pentafluorophenyl (**B**) and 4-nitrophenyl (**C**) esters of 4-(azidosulfonyl)-benzoic acid (**A**).

*Pentafluorophenyl* (**B**) *and 4-nitrophenyl* (**C**) *esters of 4-(azidosulfonyl)-benzoic acid*. 4-Carboxybenzenesulfonyl azide **A** (1 eq., 1 g, 4.4 mmol) and pentafluorophenol (1.1 eq., 0.89 g, 4.8 mmol) were dissolved in 20 mL of dry THF and cooled on ice bath to 0°C with magnetic stirring. Ten millilitres of DCC (1.1 eq., 0.89 g, 4.8 mmol) solution in THF were added dropwise to the reaction mixture, the ice bath was removed, and the mixture was stirred overnight at ambient temperature. Afterwards, the suspension was filtered, the precipitate washed with a small volume of cold THF, and the filtrate evaporated *in vacuo* to dryness. The remaining white solid was recrystallized from ethylacetate – *n*-hexane (1:1 v/v) affording 1.07 g (62%) of the pentafluorophenyl ester **B** as colorless crystals.

$^{1}$H NMR (500 MHz, $CDCl_3$, δ, ppm): 8.13 (d, 2H, *J* = 8.2 Hz, H*7*); 8.40 (d, 2H, *J* = 8.2 Hz, H*6*). $^{13}$C (100 MHz, $CDCl_3$, δ, ppm): 127.82 (C*7*); 131.76 (C*6*); 132.31 (C*5*); 136.2, 139.24 (dm, *J* = 243 Hz, C*2*); 138.11, 141.48 (dm, *J* = 254 Hz, C*4*); 142.82, 139.24 (dm, *J* = 245 Hz, C*3*), 139.31 (C*1*), 143.54 (C*8*), 160.73 (CO). $^{19}$F NMR (282 MHz, $CDCl_3$, δ, ppm): –162.36 (dd, 2F, $J_1$ = 17.6 Hz, $J_2$ = 22 Hz, *m*-F), –157.43 (t, 1F, *J* = 22 Hz, *p*-F), –153.11 (d, 2F, *J* = 17.6 Hz, *o*-F).

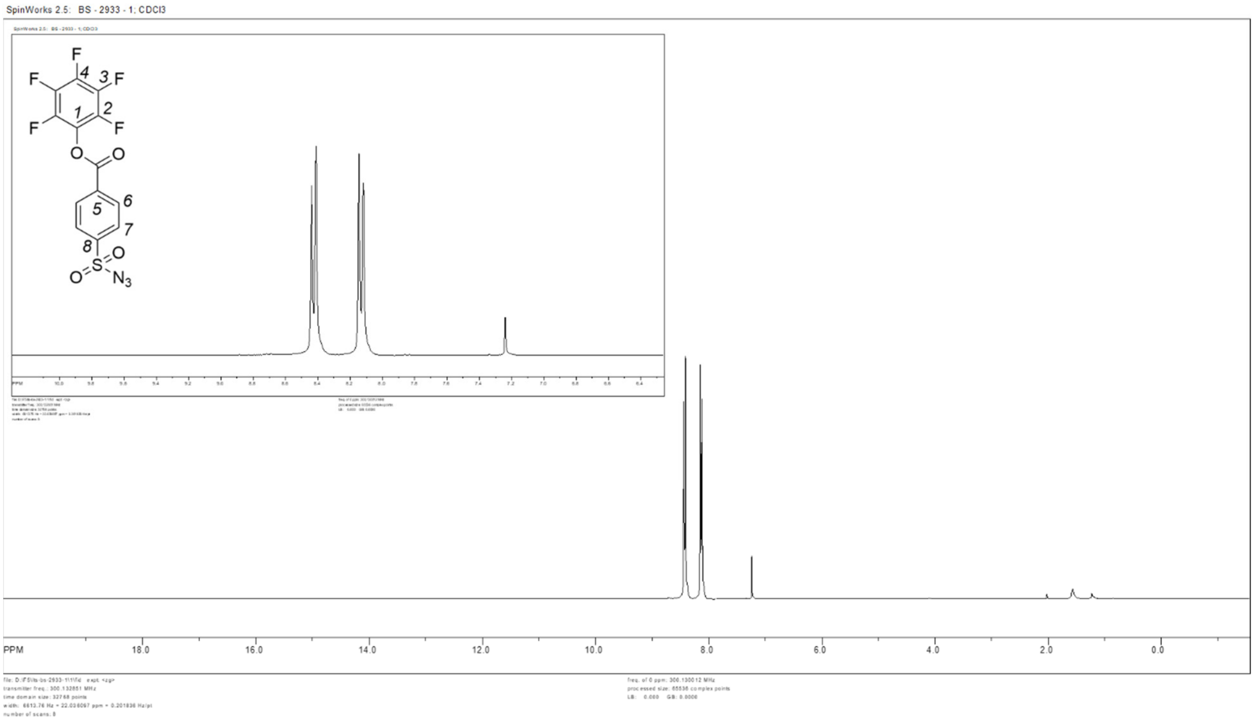


**Figure S13**. $^{1}$H NMR spectrum of pentafluorophenyl 4-(azidosulfonyl)-benzoate (**B**). Inset: expanded peak region 10.0 – 6.4 ppm.

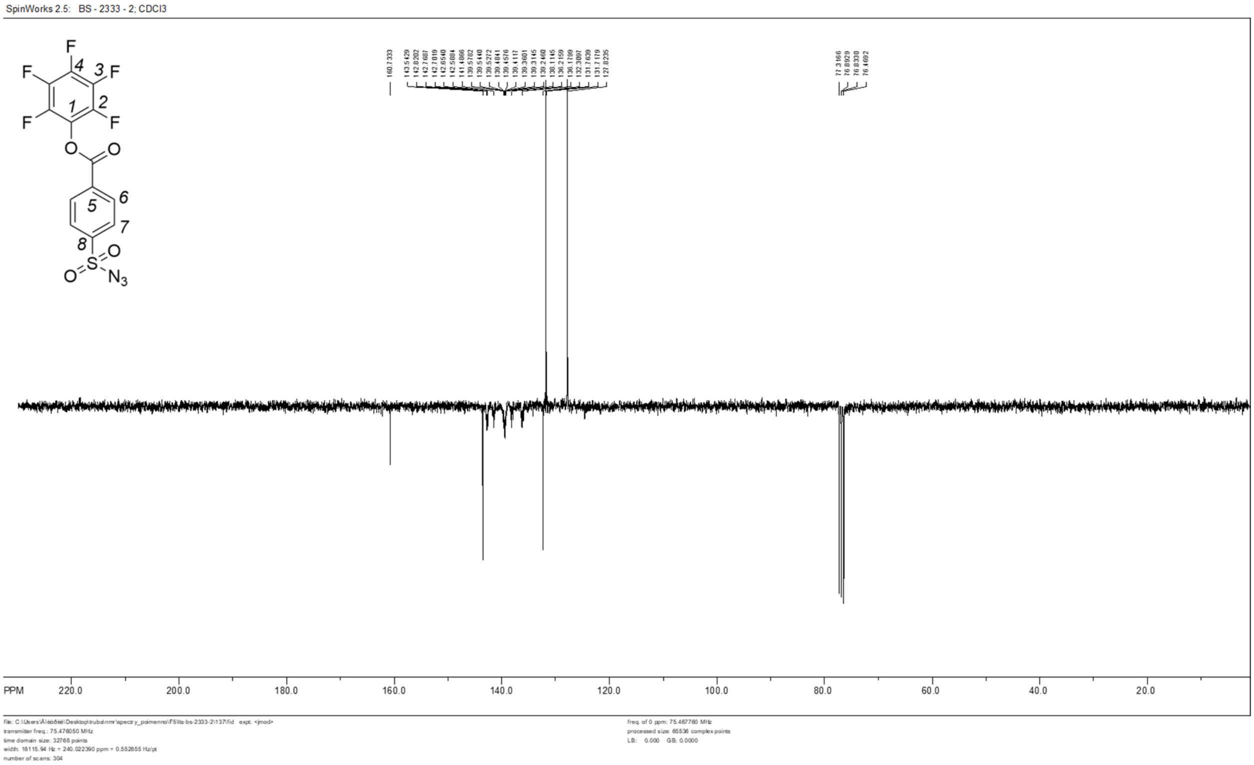


**Figure S14**. JMOD $^{13}$C NMR spectrum of pentafluorophenyl 4-(azidosulfonyl)-benzoate (**B**).

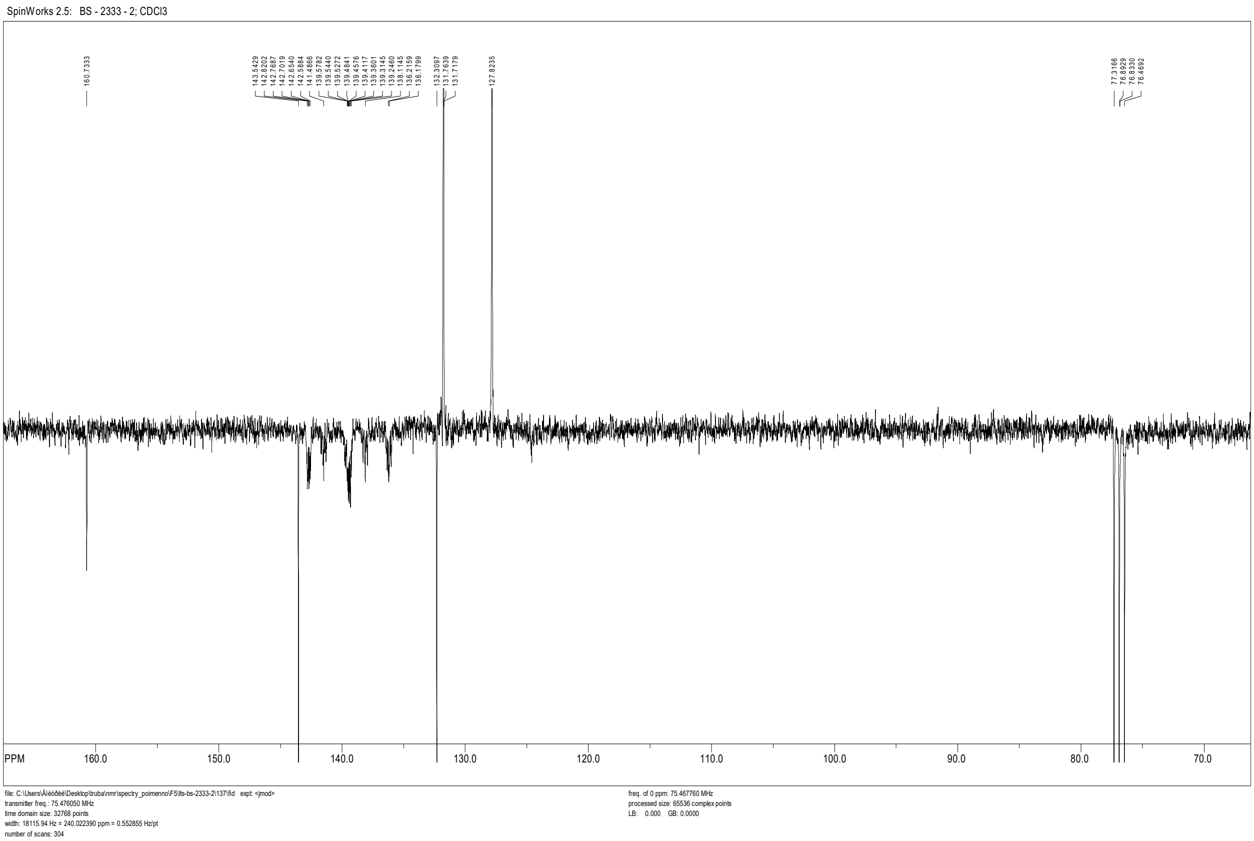


**Figure S15**. Expanded 165.0 to 70.0 ppm region of JMOD $^{13}$C NMR spectrum of pentafluorophenyl 4-(azidosulfonyl)-benzoate (**B**).

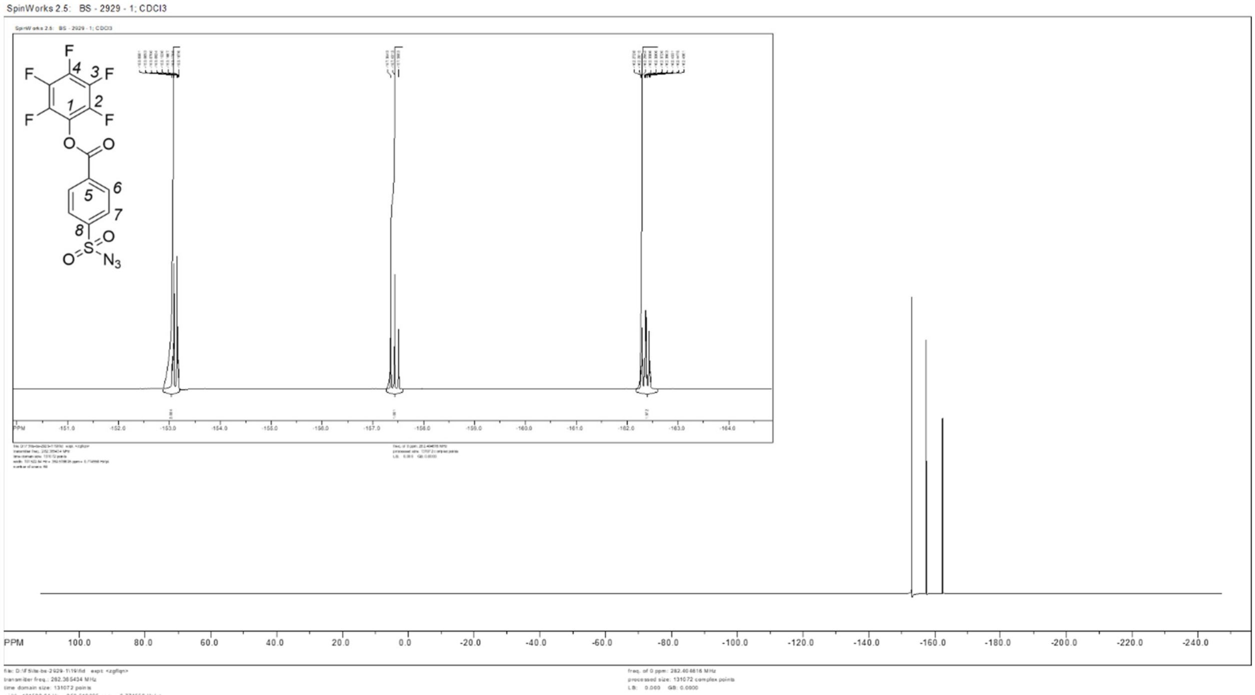


**Figure S16**. $^{19}$F NMR spectrum of pentafluorophenyl 4-(azidosulfonyl)-benzoate (**B**). Inset: expanded –151.0 to –164.0 ppm region.

4-Nitrophenyl ester of 4-carboxybenzenesulfonazide (**C**) was prepared analogously to the above from **A** (1 eq., 0.5 g, 2.2 mmol) and 4-nitrophenol (1.1 eq., 0.337 g, 2.4 mmol), providing 243 mg (32%) of the respective ester **C**.

$^{1}H$ NMR (500 MHz, $CDCl_3$, δ, ppm): 7.19 (d, 2H, *J* = 8.9 Hz, H2), 7.93 (d, 2H, *J* = 8.5 Hz, H*4*), 8.18 (broad d, 4H, *J* = 8.8 Hz, H*3* and H*1*). $^{13}C$ NMR (100 MHz, $CDCl_3$, δ, ppm): 123.04 (C2), 125.56 (C*1*), 128.68 (C*4*), 130.25 (C*3*), 135.93 (*C*CO) 139.46 ($CNO_2$), 146.36 (CO), 153.31 ($CSO_2$), 170.75 (CO).

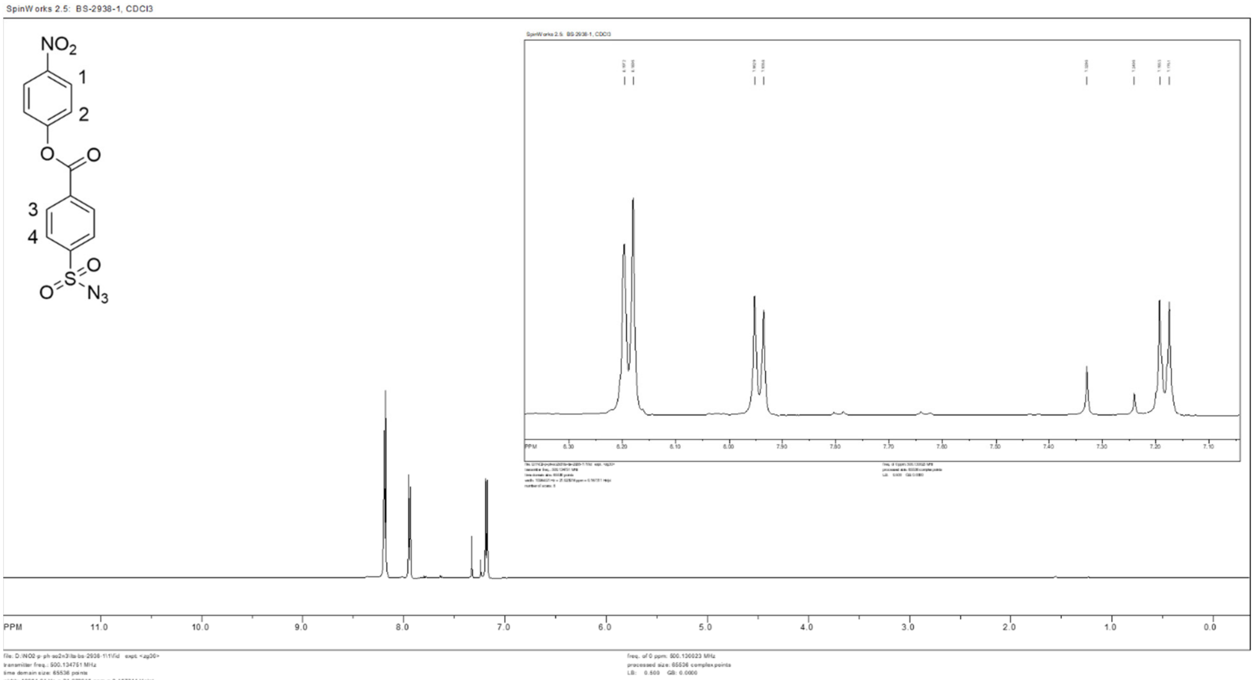


**Figure S17**. $^{1}H$ NMR spectrum of 4-nitrophenyl 4-(azidosulfonyl)-benzoate (**C**). Inset: expanded peak region 8.30 – 7.10 ppm.

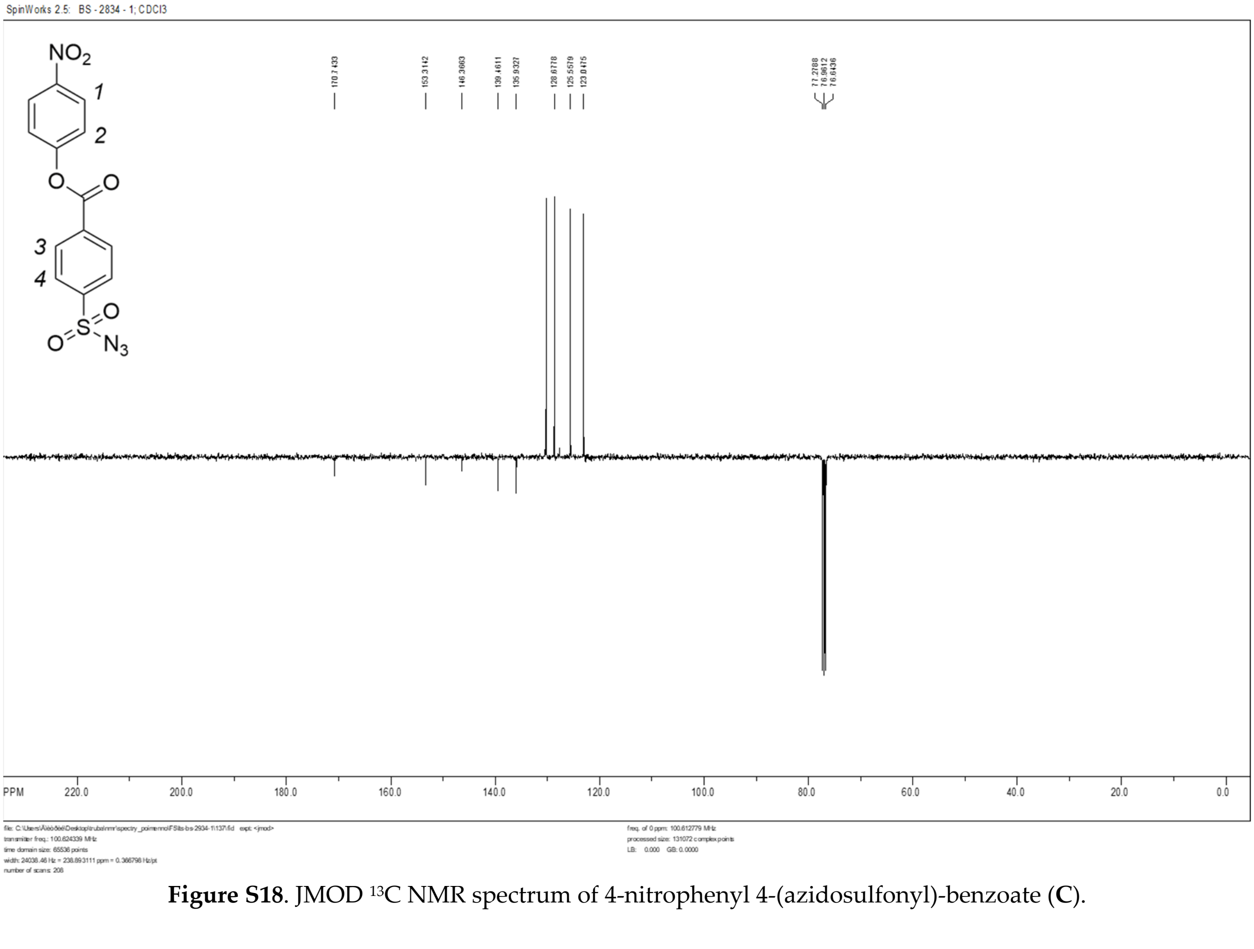


**Figure S18**. JMOD $^{13}C$ NMR spectrum of 4-nitrophenyl 4-(azidosulfonyl)-benzoate (**C**).

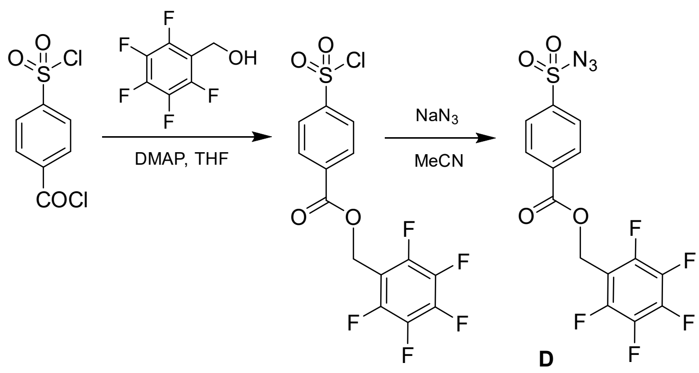


**Figure S19**. Synthesis of pentafluorobenzyl 4-(azidosulfonyl)-benzoate (**D**).

*Pentafluorobenzyl 4-(azidosulfonyl)benzoate* (**D**). To 2.39 g (10 mmol) of the available 4-(chlorosulfonyl)benzoyl chloride (CAS 7516-60-1) in 5 ml of THF, 1.98 g (10 mmol) of pentafluorobenzyl alcohol and 1.22 g (10 mmol) of DMAP were added. The reaction mixture was stirred for 5 h, then 10 ml of acetonitrile and 0.65 g (10 mmol) of $NaN_3$ were added. The reaction was further stirred for 2 days. The reaction mixture was then passed through a short layer of silica gel flashed with ethyl acetate, the volatiles were removed in vacuo, and the colorless oil was recrystallized from petroleum ether. The yield of white crystalline ester **D** was 3.0 g (75%). $^1$H NMR (300 MHz, $CDCl_3$, δ, ppm): 8.22, 8.00 (ddd, 2×2H, C*H*, $^4$ $^4J_{CH}$ = 1.5 Hz; 1.5 Hz; 8.5 Hz); 5.49 (s, 2H, C*H*$_2$). $^{13}$C NMR (75 MHz, $CDCl_3$, δ, ppm): 163.8 (*C*O); 147.4, 143.9 (dm, $^1J_{CF}$ = 255 Hz, 2×*m*-CF); 143.6, 140.2 (dm, $^1J_{CF}$ = 255 Hz, *p*-CF), 142.34 (s, *C*SO$_2$);139.2, 135.8 (dtd, $^1J_{CF}$ = 255 Hz, $^2J_{CF}$ = 17 Hz, $^3J_{CF}$ = 4 Hz, 2×*o*-CF), 134.6 (*C*CO), 108.7 (td, $^2J_{CF}$ = 17 Hz, $^3J_{CF}$ = 4 Hz, CH$_2$*C*), 54.8 (*C*H$_2$). $^{19}$F NMR (282 MHz, $CDCl_3$, δ, ppm): −141.9 (m, 2F, *o-F*), −152.9 (t, 1F, *p-F*), −161.4 (m, 2F, *m-F*). Elemental analysis: calc. C, 41.29; H, 1.48; F, 23.32; N, 10.32; O, 15.71; S, 7.87; found C, 42.87; H, 0; F, 23.28; N, 10.73.

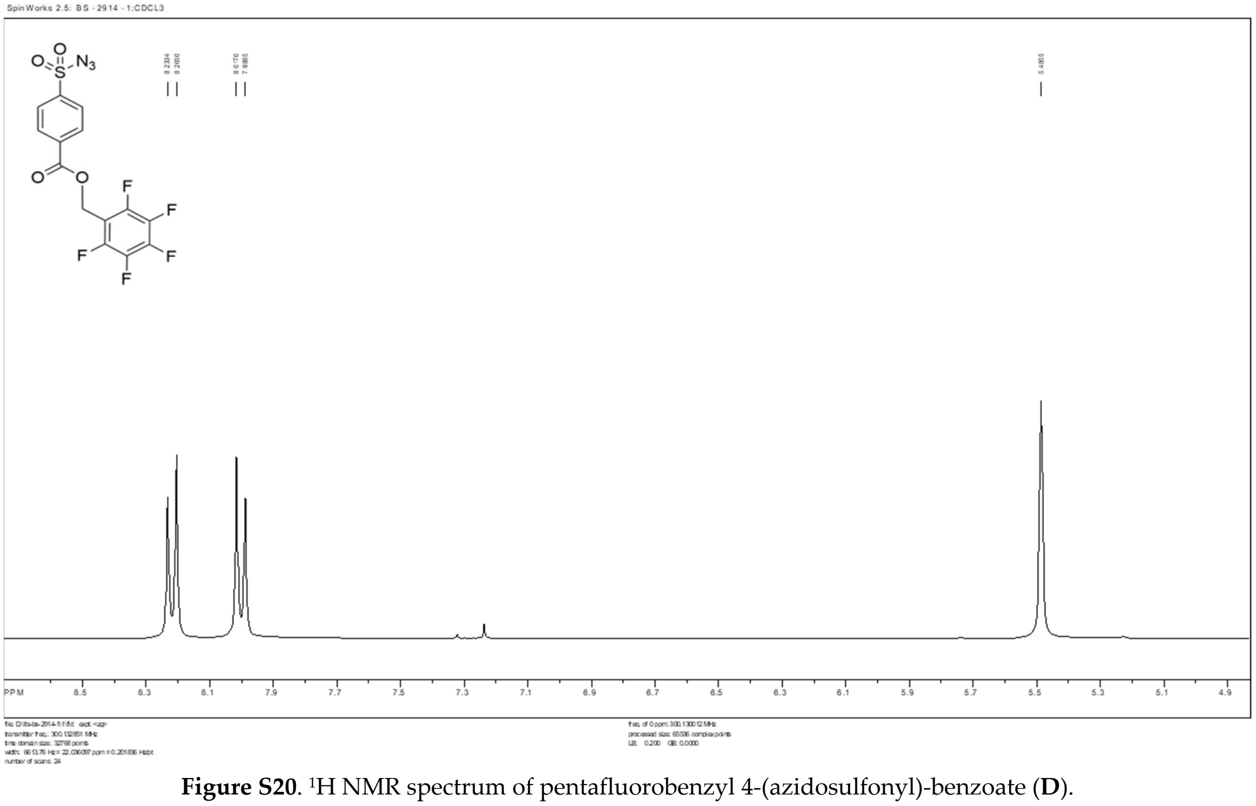


**Figure S20**. $^1$H NMR spectrum of pentafluorobenzyl 4-(azidosulfonyl)-benzoate (**D**).

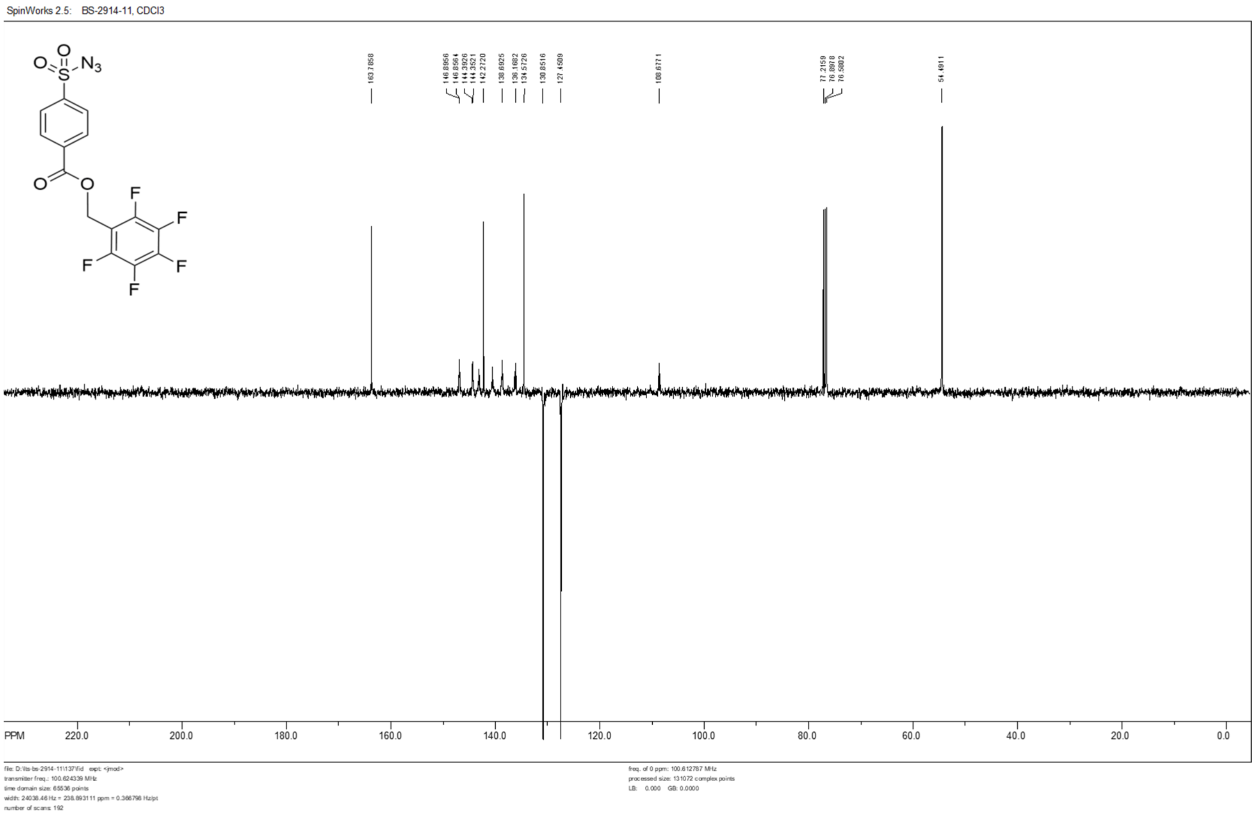


**Figure S21**. JMOD $^{13}$C NMR spectrum of pentafluorobenzyl 4-(azidosulfonyl)-benzoate (**D**).

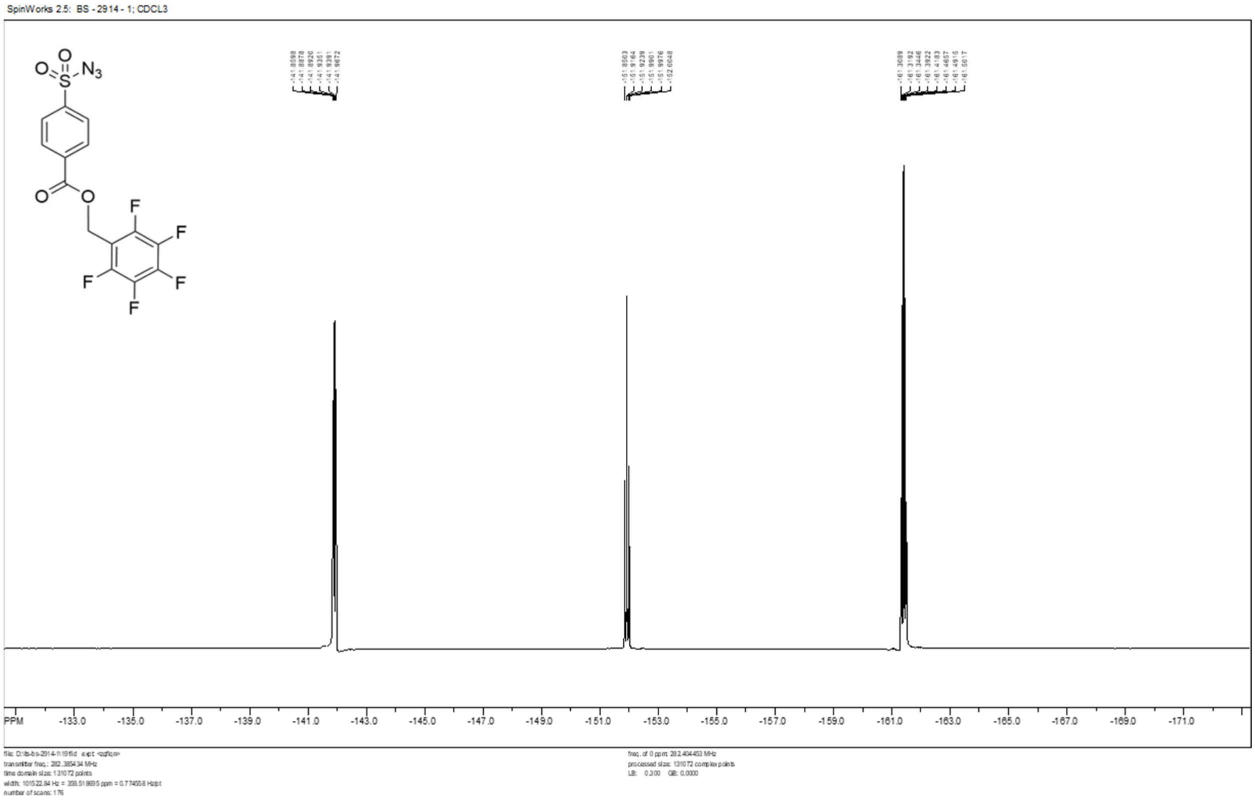


**Figure S22**. $^{19}$F NMR spectrum of pentafluorobenzyl 4-(azidosulfonyl)-benzoate (**D**).

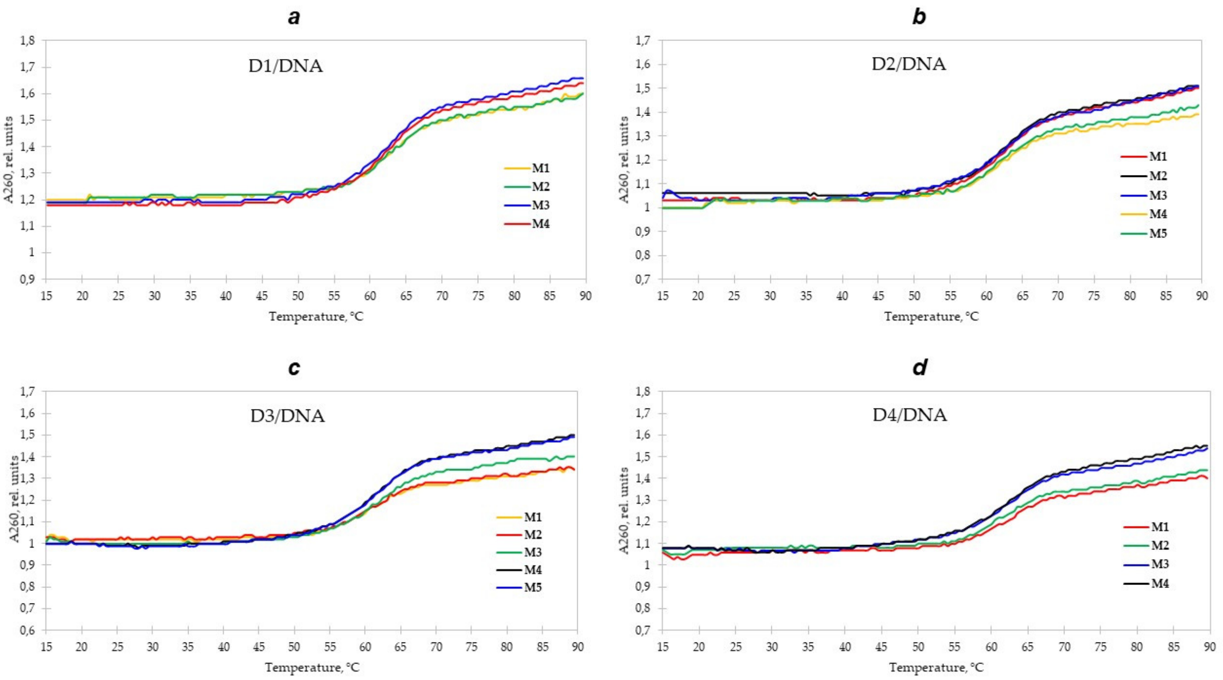


**Figure S23**. UV melting curves of the duplexes of modified oligonucleotides with DNA template: ***a***) oligonucleotide **D1** 5′-d(CTCCCAGGCTCAAA$^{\xi 1}$T); ***b***) oligonucleotide **D2 5′-**d(C$^{\xi 1}$TCCCAGGCTCAAAT); ***c***) oligonucleotide **D3 5′-**d(CTCCCAGG$^{\xi 1}$CTCAAAT); ***d***) oligonucleotide **D4** 5′-d(C$^{\xi 1}$TCCCAGGCTCAAA$^{\xi 1}$T); M1-M5 represent different measurements.

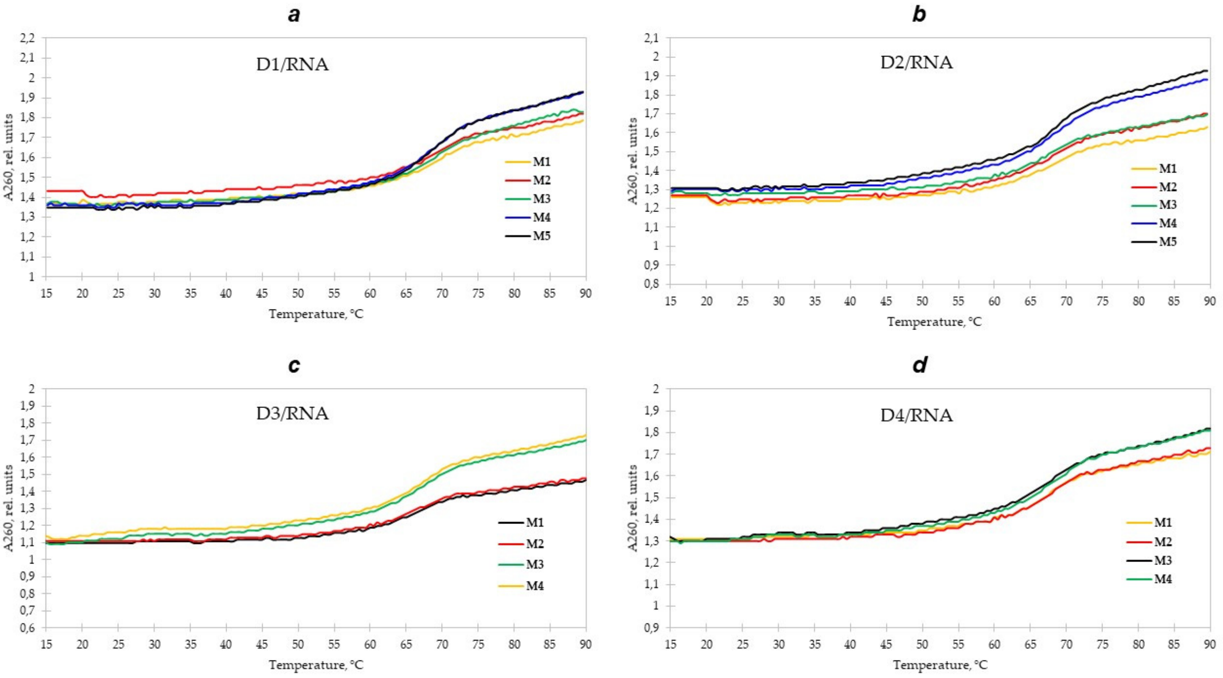


**Figure S24**. UV melting curves of the duplexes of modified oligonucleotides with RNA template: ***a***) oligonucleotide **D1** 5′-d(CTCCCAGGCTCAAA$^{\xi 1}$T); ***b***) oligonucleotide **D2 5′-**d(C$^{\xi 1}$TCCCAGGCTCAAAT); ***c***) oligonucleotide **D3 5′-**d(CTCCCAGG$^{\xi 1}$CTCAAAT); ***d***) oligonucleotide **D4 5′-**d(C$^{\xi 1}$TCCCAGGCTCAAA$^{\xi 1}$T); M1-M5 represent different measurements.
